# Can ELISPOT Be Applied to A Clinical Setting as A Diagnostic Utility for Neuroborreliosis?

**DOI:** 10.3390/cells1020153

**Published:** 2012-06-08

**Authors:** Marika Nordberg, Pia Forsberg, Dag Nyman, Barbro H. Skogman, Clara Nyberg, Jan Ernerudh, Ingvar Eliasson, Christina Ekerfelt

**Affiliations:** 1 Department of Clinical and Experimental Medicine, Infectious Medicine Faculty of Health Sciences, Linköping University, 581 83 Linköping, Sweden; Email: pia.forsberg@liu.se; 2 The Åland Borrelia Group, Åland, 22100 Mariehamn, Åland, Finland; Email: dag.nyman@aland.net (D.N.); clara.nyberg@ahs.ax (C.N.); 3 Department of Infectious Diseases, County Council of Östergötland, 581 85 Linköping, Sweden; 4 Center for Clinical Research (CKF) Dalarna, 791 36 Falun, Sweden; Email: barbro.hedinskogman@ltdalarna.se; 5 Department of Clinical and Experimental Medicine, Clinical Immunology, Linköping University, S-581 83 Linköping, Sweden; Email: jan.ernerudh@lio.se (J.E.); christina.ekerfelt@liu.se (C.E.); 6 Department of Clinical Immunology and Transfusion Medicine, County Council of Östergötland, 581 85 Linköping, Sweden; 7 Department of Laboratory Medicine, NÄL, 461 85 Trollhättan, Sweden; Email: ingvar.eliasson@vgregion.se

**Keywords:** *Borrelia burgdorferi*, neuroborreliosis, ELISPOT, cerebrospinal fluid, diagnostic test, sensitivity, specificity

## Abstract

The aim of this prospective study was to investigate the diagnostic performance of *Borrelia (Bb)-*induced interferon (IFN)-γ secretion detected by ELISPOT modified to be feasible for clinical laboratories as a supplementary test to the laboratory diagnosis of Lyme neuroborreliosis (LNB) in an endemic setting. Between 2002 and 2004, patients with symptoms of suspected clinical LNB were included in a study conducted on the Åland islands in the Finnish archipelago, which is a hyper-endemic area for Lyme borreliosis (LB). Fourteen patients with confirmed LNB and 103 patients with non-LNB were included, and the numbers of spontaneous and *Bb*-induced IFN-γ-secreting cells were assayed by the ELISPOT test. The ELISPOT assay showed a weak diagnostic performance with a sensitivity of 36% and a specificity of 82%. The findings in this study show that this ELISPOT-assay modified to be feasible in clinical routine laboratories is not useful as a supplementary diagnostic tool in the laboratory diagnosis of patients with clinically suspected LNB.

## 1. Introduction

Lyme neuroborreliosis (LNB), caused by spirochetes of the *Borrelia burgdorferi (Bb) sensu lato* genospecies complex, is one of the disseminated forms of Lyme borreliosis (LB). The spirochetes invade the nervous system and cause meningitis, inflammation of the cranial or peripheral nerves, and on rare occasions, encephalitis [1,2] .

Laboratory diagnosis of LNB, which is necessary to confirm the diagnosis, is plagued with well-known difficulties [3,4,5]. Spirochetes are difficult to grow [6] and the sensitivity of the culture and polymerase chain reaction (PCR) is low in cerebrospinal fluid (CSF) [7]. Lacking a gold standard, the presence of *Bb*-specific antibodies in the CSF with evidence of intrathecal production is recommended for diagnosis of LNB [3]. This method has its limitations, considering that intrathecal antibodies may persist for years after resolution of the infection [8,9]. Further, intrathecal *Bb* antibody production may be absent for some weeks in the initial phase of LNB [10] and several seronegative patients with LB and LNB have been reported previously [11,12,13,14,15]. In addition, false positive IgM antibody findings due to cross-reactivity sometimes occur [16,17]. A two-step (two-tier) approach has been recommended in Europe and the United States [6,18]. When a positive screening result in serum is found, an immunoblot or other second test is performed as a confirmatory test [6,19]. In suspected cases of LNB, the examination of paired CSF and serum samples makes it possible to determine the presence of intrathecally-produced anti-*Bb* antibodies by calculating a specific anti-*Bb* antibody index [1,5]. However, the sensitivity of the anti-*Bb* antibody index varies from 55–80% [4,20,21,22,23,24,25]. Today, a number of different serological assays are available, for example, antigen mixtures of the *Bb* whole-cell sonicate, purified flagellar components, recombinant antigens and the synthetic C6 peptide derived from the VlsE sequence [26,27]. 

Overall, although diagnostic tests based on *Bb*-antibodies have been improved, there is a clear need for supplementary laboratory tests in selected cases of suspected LNB. Moreover, in areas where LB is highly endemic, a diagnostic test with high specificity, which could distinguish true negative cases from positive ones, would be helpful. Further, a test robust enough for use locally in clinical routine laboratories would be preferable.

Previous studies have noted that a T cell proliferative response may occur in LB [15], and it was suggested that T cell proliferative responses could be used as a diagnostic tool in selected cases of LNB [28]. Subsequent studies on *Bb*-induced *in vitro* cytokine secretion in mononuclear cells from patients with LB have unambiguously shown predominating Th1-responses [29,30,31,32,33,34], most pronounced within the target organ *i.e.*, in the CSF or joint fluid [35,36,37,38,39]. We have previously reported pilot data from smaller studies on *Bb*-induced IFN-γ-secretion in LNB-patients and patients with other diagnoses, suggesting a fairly good diagnostic performance of ELISPOT-detected *Bb*-induced IFN-γ-secretion for LNB in CSF but not in blood [30]. Additionally, an ELISPOT (T-SPOT.TB) technique has been successfully used in the diagnosis of *M. tuberculosis* infection [40,41] .

The aim of this study was to investigate the diagnostic performance of *Bb*-induced IFN-γ-secretion detected by ELISPOT modified to be feasible for local clinical laboratories as a supplementary test to the laboratory diagnosis of LNB used as part of a clinical routine in a large prospective study in an endemic setting where clinically suspected cases are admitted. 

## 2. Results and Discussion

The current study, aiming to investigate the diagnostic performance of the modified ELISPOT-test, was carried out in a clinical setting on well-characterized LNB material collected on the Åland islands, a high-endemic area for LB, with an incidence of 1700 cases/100,000 inhabitants/year [42]. 

**Figure 1:**
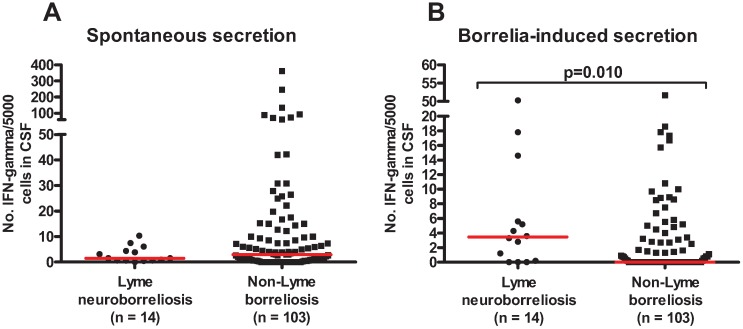
Spontaneous (**A**) and *Borrelia*-induced IFN-γ secretion (**B**) in cerebrospinal fluid from patients with Lyme neuroborreliosis and non-Lyme borreliosis. Medians are shown. The P-value represents the difference between the groups when compared using a Mann-Whitney U-test.

The numbers of spontaneous and *Bb*-induced IFN-γ-secreting cells detected by ELISPOT in LNB and non-LNB are shown in Figure 1. LNB patients showed an increased number of *Bb*-induced IFN-γ secreting cells compared with the non-LNB group (*p* = 0.010). No difference between the groups was seen regarding spontaneous secretion. When comparing spontaneous secretion with *Bb*-induced IFN-γ secretion within the groups, significantly lower spontaneous secretion was found within the LNB group (*p* = 0.017), whereas no difference was found within the non-LNB group. Further, the non-LNB group showed a strong correlation between spontaneous and *Bb*-induced IFN- γ secretion (r = 0.952, *p* < 0.0001), but no correlation was found between spontaneous and Bb-induced IFN-γ secretion in the LNB group. This latter observation is intriguing and hard to explain, and needs to be further investigated. 

**Figure 2:**
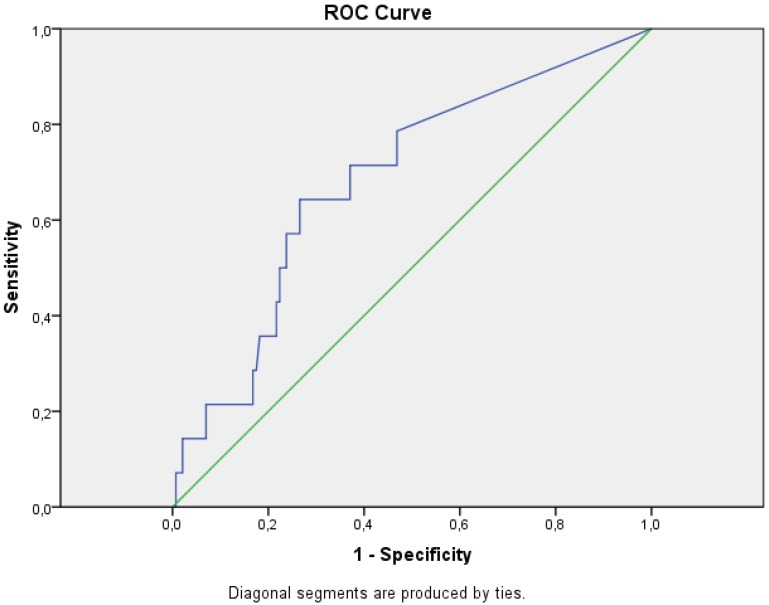
Receiver operating characteristics (ROC)-curve. The true positive rate (sensitivity) was plotted as a function of the false positive rate (1- specificity) for different cut-off points of number of *Borrelia* induced IFN-γ secreting cells.

The number of patients showing positive and negative tests for *Bb*-induced IFN-γ secreting cells in LNB and non-LNB at different cut-offs, 5 and 10 spots, are shown in Table 1. Calculated sensitivity, using the confirmed LNB (n = 14) and the non-LNB group (n = 103), yielded a sensitivity of 36% and 21% when 5 and 10 *Bb*-induced IFN-γ secreting cells were used as cut-offs for positive tests, respectively. The calculated specificity was 82% and 92% when using the cut-offs 5 and 10 spots, respectively (Table 1). The diagnostic performance is also shown in Figure 2 as an ROC curve. Furthermore, 19 of the 103 non-LNB patients, stimulated with outer surface protein (osp) enriched fraction of *Borrelia garinii* Ip90 antigen (OF) were positive in the ELISPOT test but did not have any evidence of an ongoing LB. The different diagnoses in this group are shown in Table 2A. In Table 2B, the diagnoses of the non-LNB patients with high spontaneous base-line (≥15 spots) secretion of IFN-γ are described.

**Table 1:** Diagnostic performance of ELISPOT-detected *Borrelia*-induced Interferon-γ secretion in patients with Lyme neuroborreliosis and non-Lyme neuroborreliosis, utilizing different cut-offs.

Diagnostic groups	ELISPOT cut-off ≥5 spots		ELISPOT cut-off ≥10 spots	
	Positive	Negative	Positive	Negative
Lyme neuroborreliosis (n = 14)	5 (36%)	9 (64%)	3 (21%)	11 (79%)
Non-Lyme borreliosis (n = 103)	19 (18%)	84 (82%)	8 (8%)	95 (92%)

Thus, although LNB patients, as expected, showed increased numbers of Bb-induced IFN-γ secreting cells in CSF compared with non-LNB patients [30,35,43], the assay showed weak diagnostic performance. Our previous studies suggested a stronger diagnostic performance [30,35]. However, these studies were conducted in areas with lower seroprevalence (5.8%) for LB, the annual incidence in southern Sweden being 69 cases per 100,000 inhabitants/year [44,45].

**Table 2:** (**A**) *Borrelia-*induced Non-Lyme neuroborreliosis patients that were positive in the ELISPOT (≥5 spots) assay in cerebrospinal fluid, classified into different diagnostic groups; (**B**) Non-Lyme neuroborreliosis patients with spontaneous secretion of 15 spots or more, classified into different diagnostic groups.

**Diagnostic groups Non-Lyme neuroborreliosis**	**Number (n = 19)**
Pain/myalgia/arthralgia/fatigue	n = 6
Other infection Pyelonephritis Unspecific infection Tendinitis Tooth infection	n = 4
Other CNS disease Alzheimer’s disease Neuropathy Sensibility disorder	n = 3
Autoimmune Collagenosis Rheumatoid arthritis	n = 2
Inflammatory CNS disease Tick-borne encephalitis	n = 2
Endocrine Hypothyroidism	n = 1
Lymphoma	n = 1
(**A**)	
**Diagnostic groups Non-Lyme neuroborreliosis**	**Number (n = 23)**
Pain/myalgia/arthralgia/fatigue	n = 8
Autoimmune Mb Reiter Collagenosis Rheumatoid arthritis	n = 4
Other CNS disease Epilepsy Neuropathy Sensibility disorder	n = 4
Other infection Pyelonephritis Tooth infection Herpes keratitis	n = 3
Inflammatory CNS disease Tick-borne encephalitis	n = 2
Lymphoma	n = 1
Unknown diagnosis	n = 1
(**B**)	

The non-LNB patients in this study had no evidence of an ongoing *Bb*-infection in the CNS, but some of them were seropositive for *Bb* in blood, indicating an earlier infection with *Bb*. Surprisingly, 19 of the 103 non-LNB patients were positive in the ELISPOT test. This is difficult to explain, since they all had different diagnoses (Table 2A) with no single CNS disease that could possibly have explained this. Memory T cells are known to patrol tissues where the infection originally occurred, and they also react to antigens faster than naïve T cells. It is possible that previously generated memory cells circulating in the CSF, acting on some inflammatory trigger, could explain the high number of *Bb*-induced IFN-γ secreting cells in the non-LNB group in the current study, which was performed in this hyper-endemic area for LB. This would also have implications for using the test in low-endemic areas, since the most likely cases having a previous LNB occur in such areas, decreasing the validity of the test. A clinician working in an endemic area would want a test with a high specificity to help in finding the true negative cases, since the *Bb* antibodies reflecting an old infection could persist for years [8,9,46].

Interestingly, when considering spontaneous secretion of IFN-γ, the non-LNB patients seemed to have higher secretion compared to the LNB patients (Figure 1). Investigating the different diagnoses in the non-LNB group (Table 2B) reveals a variety of diagnoses, and as such are not able to not explain this outcome.

One important aspect to consider is the choice of antigen. Here we used OF, *Borrelia garinii* Ip90 [47]. This decision was based on previous experience showing the ability of this antigen to discriminate LNB from controls [30], induce augmented intrathecal IFN- γ secretion [48], demonstrate compartmentalization of IFN-γ response [35] and induce a strong CSF response in adults [49] and in children [43]. We also identified a set of potential T cell epitopes from OspA (which is one of the major antigens in OF), although their diagnostic relevance has not been established [50]. Thus, taken together, the chosen antigen is a logical choice in LNB, although the possibility that other antigens or a combination of antigens could have led to different results cannot be excluded.

The current study aimed to develop a supplementary diagnostic test, feasible for clinical laboratories. Therefore, instructions were developed based on the idea that the CSF should simply be centrifuged, the supernatant removed and the pellet of cells resuspended in culture medium. A cut-off value for choosing single or duplicate wells for the test should be based on a routine cell count of CSF. This is the rationale for why different numbers of cells were assayed for the different patients, and the spot counts mathematically adjusted to 5000 cells/well. It cannot be excluded that this strategy affected the result since the relation between the *in vitro* IFN-γ response to *Bb* in mononuclear cells and the number of cells per well not necessarily is linear. However, since the non-LNB patients in general had a lower number of cells, and thus would have had less optimal culturing conditions, they would in turn have generated lower numbers of IFN-γ secreting cells. Thus, the low number of cells in the non-LNB group probably does not explain the weak diagnostic performance of the test, which mainly was due to the high number of non-LNB patients showing positive test. Regression analysis of the uncorrected cell count/well *versus* stimulated and unstimulated IFN-γ production in the diagnostic groups was performed. The LNB group’s spontaneous secretion showed a significant association with the cell count with the coefficient of determination R^2^ of 0.30, *p* = 0.041 and for the *Bb*-induced LNB group R^2^ was 0.44 and *p* = 0.01. Thus, there is a difference in IFN-γ production that is associated with the cell count but not entirely explained by it. 

IFN-γ, a Th1 cytokine, is an important mediator of inflammation, driven by infection or injury. This innate response exists irrespective of adaptive mechanisms and is an unspecific marker of inflammation. In the LNB-group, where the CSF-leukocyte count, another unspecific marker of inflammation, was elevated in all patients, the unstimulated IFN- γ secretion was low and a significant stimulation with *Bb* antigen was *found*. The spontaneous secretion correlated partly to the leukocyte count in this group (R^2^ = 0.30, *p* = 0.041).

The group of non-LNB is heterogeneous and a correlation of IFN-γ secretion to the leukocyte count was not found in unstimulated cells. However, in the subgroup of non-LNB with pleocytosis, a significant correlation to the leukocyte count was found, (R^2^=0.48, *p* = 0.006). This may be an unspecific stimulation by borrelial lipoprotein in the preparation.

## 3. Experimental Section

### 3.1. Material

#### 3.1.1. Patients

Between 2002 and 2004, 310 patients were admitted to Åland Central Hospital with symptoms of suspected clinical LNB *i.e.*, symptoms or signs compatible with meningitis, radiculitis, facial nerve palsy, other cranial nerve affections, pain, arthralgia, fatigue or dementia. Collection of EDTA plasma and a lumbar puncture was performed on all patients at inclusion. Of the 310 patients, 156 were included in the ELISPOT-study, since this assay could only be performed on weekdays during daytime hours. Fifteen patients were excluded from the study; six due to technical problems, six who could not be evaluated because of missing data or extensive co-morbidity or unclear diagnosis; and three who had received antibiotic therapy immediately prior to inclusion. A group of 19 patients out of the included 156 had other *Bb*-manifestations (with seroconversion or increasing levels of serum *Bb* antibodies) but without central nervous system (CNS) involvement (non-CNS *Bb* infection); no CSF lymphocytic pleocytosis, no intrathecal production of specific IgG antibodies, and no blood-brain-barrier (BBB) damage. The occurrence of BBB damage was estimated by calculation of the CSF/serum albumin ratio. A normal age-correlated proportion indicated intact BBB [51]. These patients were not included in the statistical evaluation since they did not show any evidence of infection in the CNS. Furthermore, five patients diagnosed with probable LNB were excluded from the statistical evaluation since we wanted to base the calculations on a “true” LNB diagnostic group (Table 3). Thus, the study-group (n = 117) used in the calculations for the diagnostic performance of the ELISPOT test consisted of 65 women and 52 men (median 58 years, range: 6–87 years).

Fourteen patients were diagnosed as confirmed LNB, five male and nine female (6–87 years of age, median 58.5 years) (Table 4). All 14 patients with confirmed LNB were followed prospectively by phone interview and/or by clinical examination by a physician (after 2 weeks, 3, 6 and 12 months). If symptoms persisted, or as otherwise needed, additional check-ups were carried out. Patients with LNB were treated according to the local recommendations [52] with antibiotics, two weeks with ceftriaxone 2 g × 1 i.v. followed by three months with amoxicillin 500 mg × 3 p.o. In cases of known allergy, or if adverse reactions occurred, doxycycline 100 mg × 2 was used instead of amoxicillin. All treatments were instituted after the sample collection was performed. In the LNB group, all but three (two of these could not be evaluated due to missing data) had recovered four months after treatment (Table 4). One patient, (no. 4, Table 4), had no CSF pleocytosis at inclusion, but developed pleocytosis (6 × 10^6^ MNC /L) one week later.

**Table 3:** Case definitions of Lymeneuroborreliosis.

Confirmed	Probable
Clinical findings compatible with disease CSF lymphocytic pleocytosis ≥5 MNC /µL Intrathecal production of specific anti-*Borrelia* IgG antibodies	Clinical findings compatible with disease Adequate response to therapy Detectable seroconversion *or* increasing titers of specific anti-*Borrelia* IgG antibodies in serum CSF lymphocytic pleocytosis ≥5 MNC/µL *or* intrathecal production of specific anti-*Borrelia* IgG antibodies *or* significant damage to BBB

CSF = cerebrospinal fluid; BBB = blood-brain-barrier; MNC = mononuclear leucocytes; IgG = immunoglobulin G.

The remaining 103 patients (non-LNB) in the study group (47 men, 56 women, median age: 58 years, range 6–87 years), did not suffer from any proven ongoing *Bb* infection; showed absence of intrathecal production of specific IgG antibodies, no seroconversion or increasing levels of serum *Bb* antibodies. In the non-LNB group fourteen patients had pleocytosis, which was compatible with another diagnosis, *i.e.*, tick-borne encephalitis (n = 4), other CNS disease (n = 4, *i.e.*, multiple sclerosis, dizziness, numbness), orthopedic diagnosis (n = 3, *i.e.*, spinal stenosis, spondylarthritis), hypothyroidism (n = 1), tonsillitis (n = 1) and unknown diagnosis (n = 1).

The remaining 89 non-LNB patients had no pleocytosis and had the following diagnoses or symptoms; pain/myalgia/arthralgia/fatigue (n = 33), other CNS disease (n = 18, *i.e.*, epilepsy, Parkinson’s disease, fibromyalgia, Alzheimer’s disease, dementia, status tick-borne encephalitis, depression, facial nerve palsy), autoimmune disorder (n = 13, *i.e.*, Sjögren’s syndrome, Crohn’s disease, collagenosis, polymyalgia rheumatica ), other infection (n = 13, *i.e.*, pneumonia, pyelonephritis, herpes keratitis, status post herpes zoster), orthopedic diagnosis (n = 7, *i.e.*, arthritis, spinal stenosis, vertebral compression), endocrine disorder (n = 3, *i.e.*, diabetes mellitus, hypothyroidism) folic acid and iron deficiency (n = 1) and lymphoma (n = 1).

### 3.2. Methods

All laboratory investigations were performed at Åland Central Hospital laboratory. *Bb*-specific antibodies were detected in serum by Mikrogen RecomWell IgG ELISA (Martinsried, Germany) and Immunetics C6Quick ELISA C6 Borrelia kit (Boston MA, USA) with confirmation of seropositivity by Mikrogen RecomBlot immunoblot (Martinsried, Germany). Intrathecal production of specific *Bb* antibodies was measured by IDEIA Lyme neuroborreliosis kit (Lyme NB ELISA kit, K6028, Dako A/S, Glostrup, Denmark). CSF analyzed before January 2004 were analyzed by Mikrogen RecomWell. All kits were used according to the manufacturers’ instructions. 

**Table 4:** Patient characteristics and clinical findings in patients with Lyme neuroborreliosis (n = 14).

No	Sex F/M	Age Years	Symptoms on admission	Seroconversion/increasing titers of serum *Bb*-antibodies	Intrathecal *Bb-*antibody production	CSF pleocytosis≥5 MNC/µL’	BBB-damage In CSF	Duration of symptoms before treatment	Time to recovery after treatment
1	F	76	Facial nerve palsy, fatigue,	+	+	7	Pos	1 day	<4 months
			Pain (back, abdomen)	+					
2	M	57	Facial nerve palsy, radiculitis	+	+	85	Pos	1 day	<4 months
3	F	72	Meningoradiculitis	+	+	31	Neg	Missing data	Missing data
4	M	77	Pain (leg, scapula),	+	+	6	Pos	2 years	1 year
			Cognitive disturbances	+					
5	F	69	Facial nerve palsy, pain	+	+	126	Pos	1 day	2 weeks
6	M	87	Fatigue, radiculitis, pain (leg)	+	+	19	Neg	Missing data	2 weeks
7	F	59	Headache, paresis, dysarthria	+	+	31	Neg	2 weeks	3 months
8	M	58	Pain (leg)	+	+	228	Pos	3.5 months	2 weeks
9	F	51	Pain (knees, hips)	+	+	74	Pos	2 weeks	3 months
10	M	12	Facial nerve palsy	+	+	350	Neg	1 day	2.5 months
11	F	6	Facial nerve palsy	+	+	300	Pos	5 days	2 weeks
12	F	6	Facial nerve palsy	+	+	168	Pos	9 days	2 weeks
13	F	66	Facial nerve palsy	+	+	132	Pos	1 day	11 days
14	F	58	Pain (abdomen)	+	+	45	Pos	Missing data	Missing data

Sex; M = male, F = female, *Bb* = *Borrelia burgdorferi*, ab = antibodies, CSF = cerebrospinal fluid, BBB = blood-brain-barrier.

#### 3.2.1. Enumeration of Borrelia-Induced IFN-Gamma Secreting Cells

CSF was immediately put on ice and centrifuged at +4 °C for 10 min at 400 g within 1 h after lumbar puncture. The supernatant was removed and the cells were resuspended in 200 mikroL Iscove’s modification of Dulbecco’s medium (Gibco BRL, Paisley, UK) supplemented with (given as final concentrations in the medium): L-glutamine (Flow Labs, Irvine, UK) 292 mg/L; sodium bicarbonate 3.024 g/L; penicillin 50 U/mL and streptomycin 50 mikrog/mL (Flow); 100 × non-essential amino acids 10 ml/L (Flow) and 5% heat-inactivated fetal calf serum (FCS; Flow). Ten mikroL cell suspension was diluted 1:2 with Türks reagent and mononuclear cells were counted by light microscopy. The ELISPOT analysis for enumeration of *Bb* specific IFN-γ -secreting was carried out according to the instructions provided by the manufacturer (Mabtech AB, Nacka, Sweden). The method was slightly modified and optimized for IFN-γ spots as described earlier [30,35]. An outer surface protein (osp) enriched fraction, here called OF, of *Borrelia garinii* Ip90 was prepared as previously described [47], *i.e.*, not containing any viable *Borrelia garinii* cells. The OF antigen was used in a previously optimized concentration of 10 µg/mL.

Briefly, mononuclear cells from CSF were stimulated with OF and incubated in nitrocellulose-bottomed microtiter plates, coated with anti-human IFN-γ monoclonal antibodies. Wells with unstimulated cells were cultured in parallel, representing the spontaneous secretion. The secreted IFN-γ was bound to the IFN-γ antibodies, thereby causing an imprint of the individual IFN-γ -secreting cell, here referred to as spot. Visualization of the spots was accomplished by enzymatic staining, using a biotin-conjugated secondary IFN-γ antibody, enzyme-conjugated avidin and enzyme substrate. The spots were counted in a dissection microscope. CSF cells were assayed in duplicate (2 stimulated and 2 unstimulated wells) when the yield of the cells was higher than 25,000 cells, and singly (1 stimulated and 1 unstimulated) when the yield was lower than 25,000 cells. In the whole quantity of material the number of cells/well varied between 650 cells/well and 115,000 cells/well. Within the LNB group the number of cells per well varied from 2000 up to 115,000, median 65,000, and cells from the LNB patients were assayed with 5000 cells or more per well in 12/14 cases. The number of cells in the non-LNB group varied from 650 up to 35,000, median 5500. 57/103 were assayed with 5000 cells or more per well. 

#### 3.2.2. Data handling and Statistics

In the ELISPOT method the counts of spots were adjusted to 5000 cells/well for all patients. The mean of duplicates was calculated when appropriate. The number of *Bb*-induced IFN-γ secreting cells was calculated by subtracting the number of unstimulated cells from the number of OF-stimulated cells. All the spot interpretation was undertaken by one person with experience of ELISPOT.

Statistical analyses were performed using IBM SPSS Statistics 20, and graphs were created by using Graph pad prism 5. The Mann-Whitney U-test was used for comparison of spontaneous and *Bb*-induced IFN-γ secretion between the groups. Wilcoxon’s test was used for comparison of spontaneous and *Bb*-induced IFN-γ secretion within the groups. Logistic regression was used to adjust for differences in cell count/well between LNB and non-LNB. Spearman’s test was used for correlation analysis.

Receiver operating characteristics (ROC) were displayed graphically using an ROC curve. The true positive rate (sensitivity) was plotted as a function of the false positive rate (1- specificity) for different cut-off points of a parameter. Each point on the ROC curve represents a sensitivity/specificity pair corresponding to a particular decision threshold. The area under the ROC curve is a measure of how well a parameter can distinguish between two diagnostic groups (diseased/normal). ROC curves may be used to compare the diagnostic performance of two or more laboratory or diagnostic tests [53].

This study was approved by the local ethical committee (26/5 2005, Åland).

## 4. Conclusions

In conclusion, detection of *Bb*-induced IFN-γ secreting cells by an ELISPOT-assay modified to be feasible in clinical routine laboratories showed a weak diagnostic performance as a supplementary test in laboratory diagnosis of patients with clinically suspected LNB. The assay involved drawbacks as low specificity and a procedure of extrapolating the results to 5000 cells/well due to low and varying CSF cell numbers and could therefore not be of value as a diagnostic tool in endemic areas.
